# A whole new world of healing: exploring medical hypnotherapy for pediatric patients: A review

**DOI:** 10.1007/s00431-023-04983-5

**Published:** 2023-05-05

**Authors:** Vanessa Bastek, Michel van Vliet

**Affiliations:** 1 Department of Child And Adolescent Psychiatry, Klinikum Region Hannover GmbH, Wunstorf, Germany; 2 Department of Social Pediatrics, Beatrix Children’s Hospital, University Medical Center Groningen, University of Groningen, Groningen, Netherlands

**Keywords:** Hypnotherapy, Medical hypnotherapy, Pediatrics, Children, Treatment

## Abstract

This narrative review aims to unravel the potential of medical hypnotherapy for the treatment of children with a variety of diseases and symptoms. Going beyond its history and assumed neurophysiology, the chances of success for hypnotherapy will be outlined per pediatric speciality, accentuated by clinical research and experiences. Future implications and recommendations are given on extracting the positive effects of medical hypnotherapy for all pediatricians.

*Conclusion*: Medical hypnotherapy is an effective treatment for children with specified conditions such as abdominal pain or headache. Studies suggest effectiveness for other pediatric disciplines, from the first line up to third line of care. In a time in which health is defined as 'a state of complete physical, mental and social well-being’, hypnotherapy stays an underrated treatment option for children. It is a unique mind–body treatment, which true potential still needs to be unraveled.

**Table:** 

**What is Known:** *• Mind–body health techniques become a more relevant and accepted part of treatment in pediatric patients.* *• Medical hypnotherapy is an effective treatment for children with specified conditions such as functional abdominal pain.*	
**What is New:** *• Studies suggest the effectiveness of hypnotherapy in a high variety of pediatric symptoms and disease.* *• Hypnotherapy is a unique mind–body treatment which potential goes far beyond its current utilization.*

**What is Known:**

*• Mind–body health techniques become a more relevant and accepted part of treatment in pediatric patients.*

*• Medical hypnotherapy is an effective treatment for children with specified conditions such as functional abdominal pain.*

**What is New:**

*• Studies suggest the effectiveness of hypnotherapy in a high variety of pediatric symptoms and disease.*

*• Hypnotherapy is a unique mind–body treatment which potential goes far beyond its current utilization.*

## Introduction

According to the biopsychosocial model of health and illness, causes, manifestations, and outcomes of health and illness are determined by interactions between biological, psychological and social factors [1–5]. Traditionally, pediatricians focus on all these domains when treating children.

With the introduction of ‘Positive Health’ in pediatrics, which defines health as 'a state of complete physical, mental and social well-being and not merely the absence of disease or infirmity', mind–body health techniques have become an even more relevant and accepted part of treatment [6–8]. In mind–body health techniques, medical hypnotherapy is increasingly used as an effective adjunctive treatment for several symptoms and disorders [5, 9, 10].

Hypnotherapy is ‘a state of consciousness involving focused attention and reduced peripheral awareness, characterized by an enhanced capacity for responding to suggestion’ [11, 12]. This state of consciousness is also known as trance. Most people encounter trance spontaneously several times a day (e.g. daydreaming) [5]. In hypnotherapy trance is deliberately sought to create moments of high attentiveness and receptivity to suggestions [5, 12]. These positive suggestions access the subconscious mind and are used to retrain the brain to diminish symptoms [13, 14]. Hypnotherapy can be used to teach patients coping skills [108]. It helps them to develop new pain management strategies [3, 10]. It also assists patients in reprocessing important psychodynamic emotional factors contributing to their symptoms [15].

This narrative review outlines the history of medical hypnotherapy in modern medicine, its neurophysiology and the essential elements of a hypnotherapy session. Moreover, it summarizes the evidence for the effectiveness of hypnotherapy in categories of symptoms in which the treatment is nowadays being used.

### History

The use of hypnotherapy-like techniques goes back until ancient times when hypnotic features were incorporated into rituals performed by priests and healers [16]. In the Western world, Franz Anton Mesmer (1734–1815) introduced hypnotherapy into medicine. His techniques were widely criticized, as he stated they were a form of a ‘sixth sense’ and could only be experienced, not defined or explained [17]. Despite the criticism, his so-called “mesmerism” was used by several surgeons as a sole analgesic technique with very positive results [16, 18].

James Braid (1795–1860) was the first to introduce the word “hypnosis” and emphasized that hypnotherapy was based on relaxation in combination with heightened suggestibility [19]. Decades later, Emile Coué (1857–1926) stated that hypnosis was self-hypnosis: a technique that could be taught to almost every patient [19]. At the same time, the first research regarding hypnotic susceptibility in children took place, facilitated by both Liebeult and Bernheim, founders of the “School of Nancy” for hypnotherapy [16]. Due to the efforts and research of Leora Kuttner, amongst others, in the past decades, medical hypnotherapy has become a more commonly used technique in pediatric medicine [20]. However, many aspects of hypnotherapy are still not fully understood and require further evaluation and research, especially regarding the underlying neurophysiology of hypnotherapy.

## The neurophysiology of hypnosis

**Fig. 1:**
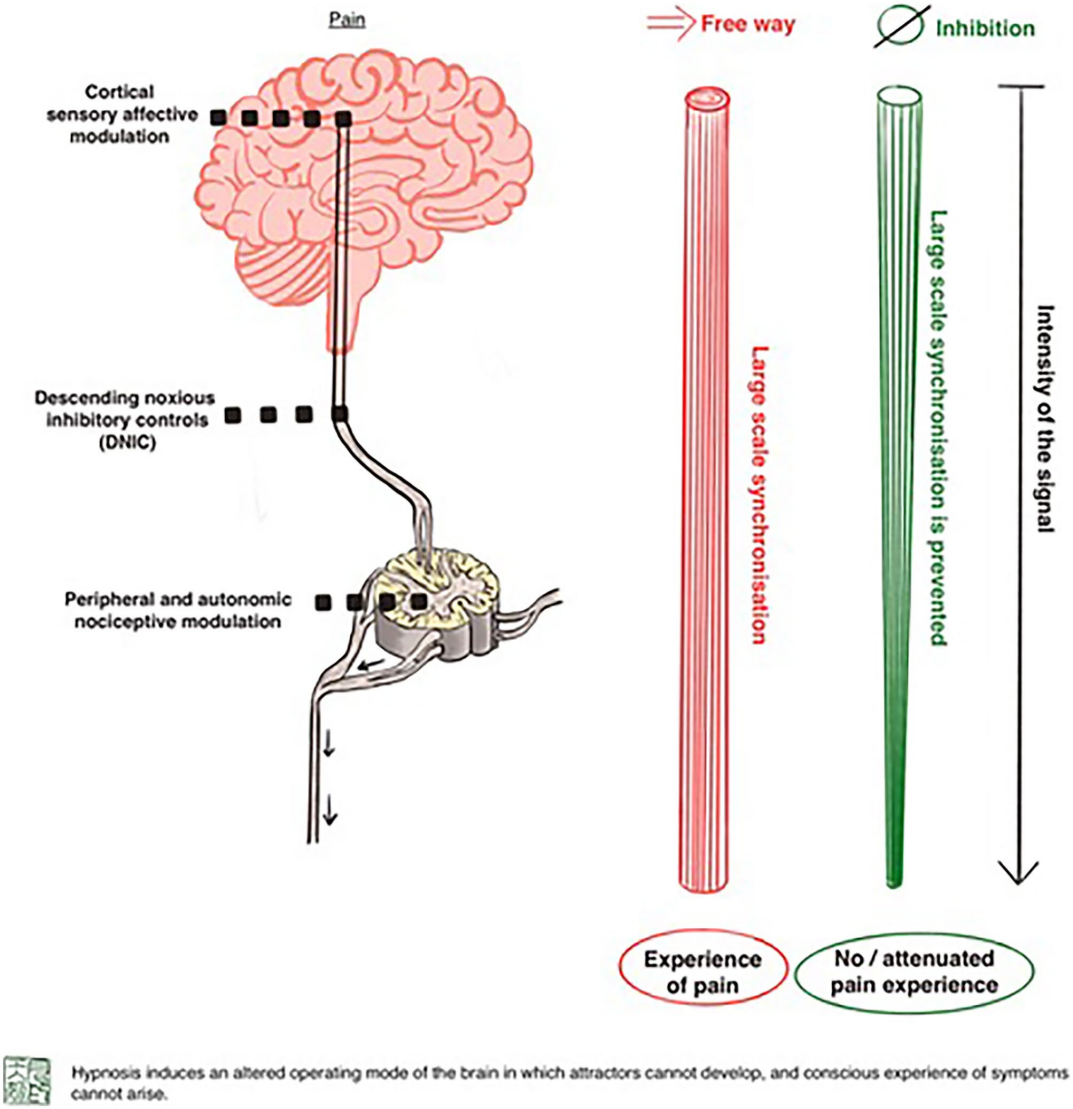
The role of attractors in the neurophysiology process of hypnosis

Keppler et al. hypothesize that hypnosis induces an altered operating mode of the brain in which so-called attractors (highly synchronized large-scale activity patterns) cannot develop and, therefore, conscious experience of symptoms cannot arise (Fig. 1) [21]. For example, to experience pain, spatially divided brain regions must be synchronized in a temporally correlated manner, and coherent large-scale activity patterns have to be formed. Hypnosis inhibits this process by modulating multiple neurocognitive mechanisms in several cortical and subcortical areas (Table 1 [5, 22]).

**Table 1:** Neurocognitive mechanisms modulated by hypnotherapy

*Hypnosis modulates pain by interfering at different neural levels and in multiple neurocognitive processes involving attention and cognitive control, appraisal processes and suggestibility*		
***Cortical influences***	Sensory-discriminative dimension of pain	Primary and secondary somatosensory cortices, thalamus and posterior insula
	Affective-motivational dimension of pain	Dorsal ACC and anterior insula
	Evaluation and appraisal of pain	Prefrontal cortex
***Spinal influences***	Influencing spinal pain transmission	Activation of descending inhibitory systems by reducing the R-III reflex*
***Autonomic nervous system***	Reducing sympathetic physiological response to stress	Regulating the HPA axis
***Peripheral nerves***	Downregulating the inflow of both A-delta and C fibers*	

*Still debated Sources [5, 19]

Despite intensive research on the working mechanisms of hypnosis, multiple questions remain (Table 2 [22–26]). Several authors have proposed that hypnosis can only be fully understood when biological, psychological and social factors are considered [5, 22–31]. They state that hypnotizability, patients’ expectancies and motivation, absorptive capacity and attitude towards hypnosis all play an important role in hypnotic responding. Likewise, the rapport between a patient and their therapist and the context in which hypnosis occurs, are important social factors in the working mechanism of hypnosis [26, 30].

**Table 2:** Neuro-imaging techniques in hypnotherapy research and biological effects of hypnotherapy

**NEURO-IMAGING TECHNIQUES IN HYPNOTHERAPY RESEARCH**	**BIOLOGICAL EFFECTS OF HYPNOTHERAPY**
Bispectral analysis	Increase in theta activity*
Single-photon emission computed tomography (spect)	Changes in gamma activity
Functional magnetic resonance imaging (fmri)	Increase/decrease in activity dorsolateral prefrontal cortex (DLPFC) and anterior cingular cortex (ACC)^a^
Positron emission tomography (pet)	Asymmetry in hemispheric activity^b^
Real-time fmri	

*Sources *[19–23]

*Associated with drowsiness, focused attention and memory functions

^a^Whether the activity in these regions increases or decreases depends on the kind of suggestions therapists give

^b^Possibly reflecting larger neurophysiological flexibility in HHSs, needed to respond to suggestions

### Interpreting results

Like all behavior and experience, hypnosis is associated with neurophysiological processes. However, several factors complicate research focusing on these neurophysiological aspects (Table 3 [5, 22, 28–32]). The essential question in interpreting the results of hypnosis research always remains: do the neurophysiological processes found only reflect hypnotic responding, or do these processes influence hypnotizability and hypnotic responding [26].

**Table 3:** Difficulties in interpreting research results

Difficulty	*Clarification*
Every aspect of hypnosis is supposed to go along with different neurophysiological processes	*Hypnosis is comprised of elements such as attentional control, concentration, mental relaxation, imagination, altered perception of the environment and disengagement of critical reasoning*
Distinguishing between hypnotic-related and task-related effects has proven to be challenging	
The inter-individual variability in the susceptibility of patients to suggestions complicates the interpretation of results	*For this reason, patients are often divided into highly susceptible to hypnosis (HHSs) or less sensitive to hypnosis (LHSs), before interpreting results*
The kind of suggestions given during hypnosis influence neurophysiological processes	*For example, suggestions for ‘letting something go’ may be associated with a decrease in frontal cortex activity, whereas in the same patient suggestions for analgesia may increase frontal cortex activity*
Different techniques are used in patients with disparate symptoms	

Sources [5, 19, 25–29]

## Elements of a hypnotherapy session

The first step of hypnosis in adults is generally the assessment of hypnotizability. However, children below the age of 14 are (almost) never refractory to hypnotic techniques, and even in the age-group between 14 and 20 years, nearly 90% of patients are hypnotizable. For this reason, most pediatric hypnotherapists start treatment without assessing their patients' hypnotic ability [3, 33].

Little research has been done regarding the most effective structure of a hypnosis session. Attentional control and mental relaxation, imagination, altered perception of the environment and disengagement of critical analytical reasoning are all elements of hypnosis which are addressed to make a hypnotherapy session effective [29, 30].

The key elements of a pediatric hypnotherapy session are represented in Table 4 [33, 34].

**Table 4:** Key elements of a hypnotherapy session

Part		Explanation	Possible techniques	Examples
1	Intake	First contact, informing about interests of the child, family situation, moments of discomfort, best individual pathway of imagination (seeing, hearing, tasting), individual goals of the child (e.g. through a questionnaire)		
2	Relaxation exercise		e.g. Breathing techniques	"[…] With each breath, a little more of that nice, relaxed feeling in your body goes exactly to those places where it’s most needed at the moment […]"
			e.g. Jacobson's relaxation technique	"Stretch your arms out in front of you… and spread your fingers… while pulling the wrist back…Keep your arms and fingers fully extended for a few seconds while you take a deep breath, as if your muscles were uncooked spaghetti strands. While you exhale, relax the muscles in your fingers, hands and arms and place them next to you, imagine as if the muscles were cooked, limp spaghetti strands. Feel the difference in muscle tension before and after the exercise"
3	Hypnotic induction	The induction of relaxation or trance state	e.g.: Magnetic fingers	[…] Put your hands out in front of you…intertwine them and slowly bend your elbows as if you are about to pray but only put your index fingers up which point to the ceiling…well done…leave a gap about 2–3 cm between your index fingers…and now look at the gap between your fingers and the tip of your fingers which soon will touch…good job [name]. So now imagine that your fingers are being magnetized…concentrate on that idea how magnetic they are right now. And once you focus on it you feel and see your fingers which start to move…you can now close your eyes when you feel comfortable And with your eyes closed you can concentrate on this gap becoming smaller and smaller.. and once you touch you can take a deep breath in and you can start to fully relax…
			e.g.: Favorite activity induction	"Tell me what you generally love to do […]. Well done. So imagine yourself now doing this favorite activity….maybe it will help when you are closing your eyes…enjoy it as much as possible…it feels better than ever before… […] Example of a boy that loves to play soccer: Imagine yourself, with your current age or a bit older, playing in your favorite soccer team while also carrying the outfit of your team….playing at your favorite position, whatever this may be. You can make yourself comfortable while you imagine that you play a match with your team…you help your team to eventually win… […]"
			e.g.: YES-Set (example of natural induction)	YES-Set (natural induction) The YES-Set essentially entails a natural form of induction/trance that helps to allow helpful/therapeutical suggestions after three rhetorical Yes-questions have been asked. For example: 1. Asking the child to sit down (1^st^ yes) 2. It’s nice that the child let’s you meet both parents (2^nd^ yes) 3. The child is wearing a beautiful red dress/sweater (3^rd^ yes) After that, helpful/therapeutical suggestions can more easily be used
4	Intervention	The technique which is relevant at that moment (e.g. reduction of fear or pain)	e.g.: Control room metaphor	"[…] I Invite you to visit a place in your brain where you can control everything that concerns your body…the central control room. And I don't know exactly how your own control room looks like…maybe there are rotary knobs, sliders, levers, …maybe it is a huge computer…or maybe it's something completely different. Later in the session the patient is able to change/regulate the knobs/levers to control his pain, for example
			e.g.: Hero story	1. Make a list of 4–6 situations in which fear has occurred (sort them from easy to difficult) 2. Ask the child to choose a hero and let him/her tell you about it 3. Ask the child to close the eyes and ask to go back to the hero in the story…and tell the child that the hero is coming for a visit so that they can together solve the problem in the easiest situation (of the list) 4. Ask the hero about the advice he has, how he thinks the situation could be handled best, and if he can do it first…together you can move to the more difficult situations
5	Posthypnotic suggestions	Suggestions which have an effect even after the hypnosis		"[…] It's good to know that you can always go back to this place and feel the pleasant feeling that you have now… whenever you want and how often you want, and the more you do this, the better it will work You can remember and save this place just like a youtube channel: you can call it the (patient name) channel. It can be a place to go to when you need it or when you want to feel comfortable, just like now…[…] "
6	Deduction	Suggestions to get the child out of the trance		"- Counting from 5 to 1…[…]" "- […] Now you’ve learned how to practice this at home or when you’re alone….when you practice, always give yourself a compliment for doing this so well… Good job. You can take all beautiful and good feelings to this special room where we have started the exercise… […]"

Patients take spoken language very literally during trance and are more susceptible to suggestions. The therapist helps the patient to ‘build’ a fantasy or daydream in which they weave suggestions wrapped up in metaphors. There are thousands of phantasies/daydreams/guided imageries, which are referred to as scripts. A chosen script has to fit the child and their interests. Suggestions are used to provide symptomatic relief, gain control of symptoms or build self-confidence. Posthypnotic suggestions are given to hint towards a favorable outcome in the (near) future. In the deduction phase, the patient is still in a trance and spoken words keep being taken very literally. The child and therapist reflect on the session, thereby addressing factors such as anxiety or relief. During all phases of a hypnotherapy session, the therapist must choose their words cautiously and wisely.

To our knowledge, no research comparing the effectiveness of different scripts has ever been performed. Most studies focusing on the effectiveness of hypnotherapy use a treatment protocol in which both the number of sessions and the structure of the sessions have been determined prior to the start of the study. However, each study uses its own treatment protocol [35, 36].

There is only one study in which a single session containing multiple inductions was compared to multiple separate sessions with one single induction: both can be effective. However, the conducted research knew considerable methodological flaws [32].

It has been demonstrated that also online hypnosis treatment can be effective in alleviating symptoms [37]. Even home-based self-exercises using a CD are non-inferior to individual hypnotherapy performed by qualified therapists [38].

## Hypnotherapy: areas of use

Medical hypnotherapy has been shown to alleviate a variety of symptoms independent of their nature or complexity [3, 5, 9, 10, 33, 35, 36]. In the following paragraphs the efficacy of medical hypnosis in procedural and acute pain, gastrointestinal, neurological and pulmonary symptoms will be discussed.

### Procedural and acute pain

Situations and places characterized by planned or unplanned invasive procedures.can be very stressful and traumatic for pediatric patients and their parents [39, 40]. Procedural comfort care is one of the methods to prevent pain and anxiety by establishing trust. In order to do so, a variety of techniques including distraction, CBT (cognitive behavioral therapy) and hypnotherapy are used [41].

Children in stressful situations are more susceptible to suggestions, an important feature of hypnotherapy. It can provide instant relief and decline of anxiety by changing patients' (perception of) discomfort. Moreover, it also enhances self-control and cooperation [42, 43].

Hypnotherapy has been extensively researched in various invasive procedures, such as needle-related procedures, lumbar punctures and bone marrow aspirations [44, 45]. Hypnotic interventions significantly reduced anxiety and pain in all these procedures [41, 44–53]. However, a Cochrane review focusing on the effect of hypnotherapy in pediatric needle-related procedures, states that many studies are of poor methodological quality. Therefore, more prospective studies are needed to confirm the results [41].

Besides reducing pain and anxiety, hypnotherapy has also been shown to reduce the need for sedatives and analgesics and the length of hospital stay. Moreover, it improves long-term outcomes and increases patients' and caretakers' satisfaction with overall treatment [52, 54, 55].

### Gastrointestinal symptoms

The effectivity of hypnotherapy in disorders of the gut-brain interaction (DGBI) has extensively been researched [35, 38, 56–73]. There is evidence that hypnotherapy modulates gastro-physiological processes such as gastric emptying, colonic motility and visceral sensitivity [56–58, 73]. Most importantly, hypnotherapy reduces gastro-intestinal symptoms in patients with DGBI [35, 38, 59]. In the Dutch guideline, hypnotherapy is recommended as a treatment for functional abdominal pain [60].

#### Irritable bowel syndrome (IBS) and functional abdominal pain (FAP)

In 2007 the results of the first randomized controlled trial showing the effectiveness of hypnotherapy in children with abdominal pain was published. At 1-year follow-up 85% of patients treated with hypnotherapy were in clinical remission compared to only 25% in the control group [35]. After a mean follow-up of 4.8 years, 68% of patients treated with hypnotherapy were still without symptoms [61]. Several years later, a multicenter trial showed that the effectiveness of home-based treatment using audio files was non-inferior to hypnotherapy provided by a therapist (success rate 62% vs 71% at 1 year follow-up) [38]. After a follow-up of 6 years, more than 80% of children were still in clinical remission [62]. Gulewitsch et al. (2017) found no difference between the effects of gut-directed hypnotherapy and unspecific hypnotherapy. However, the sample size was small, and the dropout rate was high [63].

During the COVID-19 era, hypnotherapists experimented with remote hypnotherapy using (for example) Skype. A study in adults showed a success rate of 58%, although 39% of patients thought face-to-face therapy would be more effective [64].

#### Functional esophageal disorders and symptoms.

Just like irritable bowel disease (IBS), disorders like functional dyspepsia and functional nausea are nowadays classified as disorders of gut-brain interaction (DGBI). Both visceral hypersensitivity and hypervigilance play an important role in its pathophysiology [65]. Due to that hypnotherapy is able to influence hypersensitivity and gut motility, it has been hypothesized that hypnotherapy will alleviate symptoms associated with these DGBI [66–68]. In a randomized treatment trial, the effectiveness of hypnotherapy and standard medical treatment were compared [59]. The standard treatment comprised multiple steps including lifestyle interventions, proton pump inhibitors and Iberogast. In both groups nausea decreased over time, and at the 12-month follow-up evaluation the treatment success rate was 60% in the hypnotherapy group and 55% in the standard treatment group. The authors state that hypnotherapy might effectively treat functional esophageal disorders. However, the success rate of hypnotherapy in patients with functional esophageal disorders is considerably lower than in patients with IBS.

#### Inflammatory bowel disease (IBD)

It has been postulated that hypnotherapy might result in greater resilience in patients with IBD and, possibly through immune-modulating pathways, might prolong remission time [69]. However, most studies were small and descriptive, so results have to be evaluated carefully. In adult patients with ulcerative colitis in remission, 68% of patients were still in remission one year after the start of hyponotherapy, significantly higher than the 40% in the control group [70].

Hypnotherapy has also been studied in patients with IBD in remission, who also had irritable bowel syndrome (IBS). In this study both children and adults were included. Treatment was successful in 30% of patients, a result non-superior to standard therapy [71].

Recently, the Rome foundation has recommended that ‘all chronic gastrointestinal disease should be conceptualized as gut-brain conditions with equal emphasis on the roles of mental and physical health’ [72]. Hopefully, this will result in new randomized controlled trials to evaluate the effectiveness of hypnotherapy as an adjunctive treatment for patients with both ulcerative colitis and Crohn’s disease.

### Neurological symptoms

There are just a few studies regarding medical hypnotherapy's effect on children's neurological symptoms. These studies focus mainly on headaches and functional neurological symptom disorders, including pseudo-seizures.

#### Headaches

Medical hypnosis has been studied as a treatment for both migraine and other forms of headache. However, the number of studies focusing on children and adolescents remains small [73]. Jong et al. performed a randomized controlled trial that showed the effectiveness of medical hypnotherapy and meditation in treating pediatric headaches. Most patients suffered from tension-type headache. Nine months after start of treatment 47% of patients reported a reduction of > 50% of days with headaches [74]. However, the results should be interpreted with caution as the drop-out rate was larger than 20%. Moreover, only a third of eligible patients were interested in participating in the study, which might have biased the observed results.

#### Functional neurological disorder (FND)

Hypnosis was already used as a treatment for FND by Jean-Martin Charcot in the nineteenth century. More recently, Coogle et al. described the successful treatment of a 9-year-old patient using the hypnotic magic glove technique. In their subsequent review of the literature, they describe the similarities and differences between FND, and hypnosis induced paralysis in functional neurologic imaging studies. With the premise of a shared neural pathway between hypnosis and FND, they try to explain the successful treatment of their patient [75].

Two randomized controlled trials were performed by Moene et al. [76, 77]. In the first study, they evaluated the effect of the addition of hypnosis to an already extensive treatment regimen for patients with conversion disorder of the motor type. In this small trial 83.7% of patients had improved significantly 6 months after treatment. However, the additional value of medical hypnosis was not demonstrated, and only patients older than 18 years were included [76].

In their subsequent randomized controlled trial in 2003, they demonstrated that hypnosis significantly reduced symptoms and impairments in physical, daily-life and social activities. However, in this small study, the patients were randomized between the hypnotic treatment group and waiting-list controls. Again, only patients older than 18 years were included in the trial.

So far, no studies have been conducted comparing hypnotherapy with the commonly used treatment protocol in conversion disorder/ FND.

Phansalkar et al. propose that hypnosis might be the appropriate treatment in a small part of children with functional vision disorders. However, their proposition is only based on the fact that four patients in the study by Moene et al. in 2003 were diagnosed with functional blepharospasm or ptosis [78].

### Pulmonary symptoms

In multiple studies, pediatric patients with pulmonary symptoms or disease show considerable improvement when receiving hypnotherapy [9, 13, 79–96]. Most of these studies focused on patients with asthma or chronic dyspnea.

#### Asthma

Children with severe asthma have a lower quality of life than their healthy peers, and many caretakers seek complementary treatment such as acupuncture, yoga, Tai Chi and hypnotherapy [85–87]. Even though children seem to respond particularly well, hypnotherapy has been given little attention as an adjunction treatment for asthma [79].

A preschool asthma program including hypnotherapy showed a significant improvement in symptom severity and a reduction of physician visits. Parents reported increased self-confidence with regards to self-management skills [84]. A retrospective study including 2 patients with asthma and a case-report also showed improvement in symptom severity [9, 84].

However, most studies that show the beneficial effects of hypnotherapy have been performed in adults. These studies show improvement in symptoms, bronchial hyper-responsiveness and airway obstruction as well as a decrease in the use of bronchodilators [88–90]. Until now, randomized controlled trials with objective outcome parameters performed in children are lacking [87].

#### Chronic dyspnea

Chronic dyspnea, also commonly referred to as chronic shortness of breath, can be described as ‘a subjective sensation of uncomfortable breathing comprised of various sensations of varying intensity’ existing for at least 4–8 weeks [94]. It is associated with other functional respiratory disorders such as vocal cord dysfunction, habit cough or hyperventilation [95].

Anbar et al. showed that hypnotherapy resolved all symptoms in 13 out of 16 patients (81%) and improved symptoms in the remaining 19% [82]. In all patients, of which many also had suspected co-existing conditions such as panic/ anxiety disorder or choking phobia, prior conservative treatment had failed.

A retrospective study including 126 patients with symptoms like chest pain/pressure, habit cough, hyperventilation, shortness of breath, sighing and vocal cord dysfunction, showed that symptoms improved in 80% of patients. In 26 patients, a complete resolution of symptoms occurred after 1–2 sessions [13].

A retrospective chart review of 54 patients with habit cough showed similar results: the cough dissolved in 90% of patients, in 78% even after a single hypnotherapy session [83].

## Conclusion and future perspectives

The time when health was determined as the absence of physical disease or infirmity has long passed. Nowadays, health is more and more defined as 'a state of complete physical, mental and social well-being’ [97].

This new definition demands medical professionals who ‘do not ask what disease a person has, but rather what person the disease has’, a statement made two centuries ago by Sir William Osler (1849–1919). In other words, it can be stated that using the biopsychosocial model defined by Engel in 1977 is becoming most important for today’s health care professional, even (or especially) for those working in a hospital setting. Consequently, mind–body treatment, such as medical hypnotherapy, is increasingly recognized as valuable. Hypnotherapy can teach patients coping skills and modulate subconscious beliefs that play an essential role in pain and pain management. Medical hypnotherapy is an evidence-based treatment for functional abdominal pain and is recommended as treatment of choice in the Dutch pediatric guidelines. Medical hypnotherapy is also a valuable technique to prevent and manage procedural pain and anxiety. Loeffen et al. concluded that hypnotherapy should be used in all needle procedures. Besides, they proposed that combining hypnotherapy and sedatives, virtual reality or nitrous oxide, should be the focus of future studies [98].

So far, for many other symptoms and diseases, such as neurological or pulmonary symptoms, the highest level of evidence consists of case-reports and methodologically weakly carried out retrospective studies. More randomized controlled trials are needed to unravel the true potential of hypnotherapy in these symptoms and diseases.

Even though medical hypnotherapy is progressively being used in the western medical world, its potential goes far beyond its current utilization.

## Abbreviations

CBT: Cognitive behavioral therapy
FAP: Functional abdominal pain
FND: Functional neurological disorder
DGBI: Disorder of gut-brain interaction
IBD: Inflammatory bowel disease
IBS: Irritable bowel syndrome
